# Identification of key metabolism-related genes and pathways in spontaneous preterm birth: combining bioinformatic analysis and machine learning

**DOI:** 10.3389/fendo.2024.1440436

**Published:** 2024-08-20

**Authors:** Wenqi Lv, Han Xie, Shengyu Wu, Jiaqi Dong, Yuanhui Jia, Hao Ying

**Affiliations:** ^1^ Department of Obstetrics, Shanghai First Maternity and Infant Hospital, School of Medicine, Tongji University, Shanghai Key Laboratory of Maternal Fetal Medicine, Shanghai Institute of Maternal-Fetal Medicine and Gynecologic Oncology, Shanghai, China; ^2^ Department of Clinical Medicine, Shanghai First Maternity and Infant Hospital, School of Medicine, Tongji University, Shanghai Key Laboratory of Maternal Fetal Medicine, Shanghai Institute of Maternal-Fetal Medicine and Gynecologic Oncology, Shanghai, sChina

**Keywords:** spontaneous preterm birth, metabolism, bioinformatics, machine learning, placenta, pregnancy

## Abstract

**Background:**

Spontaneous preterm birth (sPTB) is a global disease that is a leading cause of death in neonates and children younger than 5 years of age. However, the etiology of sPTB remains poorly understood. Recent evidence has shown a strong association between metabolic disorders and sPTB. To determine the metabolic alterations in sPTB patients, we used various bioinformatics methods to analyze the abnormal changes in metabolic pathways in the preterm placenta via existing datasets.

**Methods:**

In this study, we integrated two datasets (GSE203507 and GSE174415) from the NCBI GEO database for the following analysis. We utilized the “Deseq2” R package and WGCNA for differentially expressed genes (DEGs) analysis; the identified DEGs were subsequently compared with metabolism-related genes. To identify the altered metabolism-related pathways and hub genes in sPTB patients, we performed multiple functional enrichment analysis and applied three machine learning algorithms, LASSO, SVM-RFE, and RF, with the hub genes that were verified by immunohistochemistry. Additionally, we conducted single-sample gene set enrichment analysis to assess immune infiltration in the placenta.

**Results:**

We identified 228 sPTB-related DEGs that were enriched in pathways such as arachidonic acid and glutathione metabolism. A total of 3 metabolism-related hub genes, namely, ANPEP, CKMT1B, and PLA2G4A, were identified and validated in external datasets and experiments. A nomogram model was developed and evaluated with 3 hub genes; the model could reliably distinguish sPTB patients and term labor patients with an area under the curve (AUC) > 0.75 for both the training and validation sets. Immune infiltration analysis revealed immune dysregulation in sPTB patients.

**Conclusion:**

Three potential hub genes that influence the occurrence of sPTB through shadow participation in placental metabolism were identified; these results provide a new perspective for the development and targeting of treatments for sPTB.

## 1 Introduction

Preterm birth, defined as birth occurring before 37 weeks of gestation clinically, is the leading cause of neonatal morbidity and mortality (1). According to the World Health Organization (WHO), the incidence of preterm birth worldwide is 5-18%, with spontaneous preterm birth (sPTB) accounting for approximately 70% of preterm births (2). Although the survival of preterm infants has improved significantly because of technological advances, short-term complications attributed to the immaturity of multiple organ systems, such as acute respiratory distress syndrome, necrotizing enterocolitis, intracerebral hemorrhage, cerebral palsy (3), and long-term complications such as cardiovascular and metabolic disorders (4, 5), still threatened the health of offspring. Since the mechanism of term/preterm labor is not fully understood, there is a lack of effective methods for preventing and treating preterm labor.

Multiple factors such as environmental exposure, genetic factors (6), stress, infection (3), etc., contribute to the individual or community origin and development of sPTB. The placenta is a medium for the exchange of all nutrients, gases, and even fetal waste products between mothers and children throughout pregnancy (7). As a medium of communication between the mother and fetus, the placenta can strongly reflect the influence of internal and external factors on pregnancy. The appropriate formation of villi and vascular structures and the stability of physiological function in the placenta ensure a healthy and successful pregnancy; otherwise, several pregnancy and fetal complications may occur, such as preeclampsia (PE), fetal growth restriction (FGR) (8), and spontaneous preterm birth (9).

In sPTB, several functional disruptions, including apoptosis (10), senescence (11), and metabolic disorders (12), are observed in the placenta. These disruptions may contribute to the progression of pathological processes. Harmonious and appropriate maternal-fetal metabolic communication guarantees a healthy fetal environment and sufficient resources to grow until parturition (13). Increasing evidence has demonstrated the important role of placental metabolism disorders in sPTB. Several transcriptomic analyses of the placenta have revealed dysregulated metabolism-related signaling (e.g., glucose and arachidonic metabolism) in sPTB patients (14–16). Moreover, some researchers have experimentally verified that disorders in amino acid (17), iron (18), lipid and fatty acid (12, 19, 20) metabolism are associated with sPTB. Additionally, arachidonic metabolic disorders can lead to lipid peroxidation and subsequently induce placental oxidative stress (21). However, existing theories cannot thoroughly explain the specific effects of metabolism on sPTB. With few studies focused on the transcriptional regulation of metabolism, we integrated existing transcriptome data to identify pathways and key genes associated with sPTB.

sPTB is thought to at least share some characteristics associated with term labor (TL) (3). A new perspective regards the initiation of preterm labor as a syndrome of multiple pathologic processes. However, what exactly promotes the early occurrence of labor in preterm birth has not yet been elucidated. Therefore, by selecting women with TL as a control group, we integrated transcriptomic data of human placentas obtained from women with sPTB (gestational age between 28 + ^0^ and 36 + ^6^ weeks) from the Gene Expression Omnibus (GEO) database to better understand the pathogenesis of sPTB. We combined bioinformatics analysis and machine learning (ML) to screen and identify altered metabolism-related pathways and hub genes in sPTB, which could be possible effective therapeutic targets in the future.

## 2 Materials and methods

### 2.1 Recruitment of participants and sample collection

The placenta was obtained from patients in 3 groups: the (1) spontaneous preterm birth (gestational age between 28 + ^0^ and 36 + ^6^ weeks, sPTB) (1); (2) spontaneous term labor (gestational age between 37 + ^0^ and 40 + ^6^ weeks, TL); and (3) elective preterm cesarean delivery due to vasa previa, fetal distress and so on(aka preterm non-labor, PNL) groups in Shanghai First Maternity and Infant Hospital, Tongji University School of Medicine (see **Supplementary Table 4** for the clinical characteristics of patients). Patients with any other complications, such as preeclampsia, fetal growth restriction, or gestational diabetes, were excluded from this study. The study was approved by the Ethics Committee of Shanghai First Maternity and Infant Hospital (KS22305), and all the methods and operation were executed according to the relevant guidelines and regulations.

### 2.2 Dataset retrieval

All the datasets we analyzed in this study were obtained from the National Centre for Biotechnology Information Gene Expression Omnibus (NCBI GEO, http://www.ncbi.nlm.nih.gov/geo) (22), using “Preterm” or “Spontaneous preterm” as keywords. The samples in the dataset fulfilled the following criteria: (1) the species of samples was HOMO Sapiens; (2) all the samples were obtained from placenta; (3) the dataset included both sPTB (gestational age between 28 + ^0^ and 36 + ^6^ weeks) and TL (gestational age between 37 + ^0^ and 40 + ^6^ weeks) groups; and (4) both sPTB and TL samples were collected from patients who underwent spontaneous (preterm) labor. Based on these criteria, we acquired 4 GEO datasets, two of which served as training sets (GSE203507 and GSE174415) and contained 21 samples from each group, while the others served as validation sets (GSE18809 and GSE120480). The details of the datasets are shown in **Supplementary Table 1**.

### 2.3 Identification of differentially expressed genes with batch effect removal

We merged GSE203507 and GSE174415 and utilized the ComBat_seq function of the R package “sva” to remove batch effects. Significantly differentially expressed genes (DEGs) were identified with the R package “DESeq2” under the criteria of p-adjustment < 0.05 and log2 | fold-change (FC) | >0.5. Subsequently, DEGs were visualized using the R packages “pheatmap” and “ggplot2”.

### 2.4 Weighted correlation network analysis

Weighted correlation network analysis (WGCNA) can identify clusters (modules) of highly correlated genes and relate the characteristic module genes with external sample traits (23). In this study, we used it to assess the relationship between module eigengene and sPTB. We extracted genes whose median absolute deviation (MAD) was in the top 75% for “WGCNA” analysis using the R package “WGCNA”. The algorithm goodSamplesGenes was used to ensure that there were no abnormal samples or genes. Moreover, we used the hclust function to construct a cluster dendrogram and determine whether there were outlier samples, and eventually, we removed two samples whose heights were greater than 70. After the soft threshold was determined to be 8, with a minimum of 30 genes per module, we obtained modules of similar coexpressed genes and applied Pearson correlation analysis to define the association between modules and disease phenotypes.

### 2.5 Functional enrichment analysis

To investigate the potential functions of the genes, gene set enrichment analysis (GSEA) (24), Gene Ontology (GO) (25, 26), Kyoto Encyclopedia of Genes and Genomes (KEGG) (27) pathway were performed, enabling us to understand the biological functions of these genes in a comprehensive and multifaceted way. Then we conducted CNBplot (28) analyses to visualize the possible links between these functions at the genetic level. The analyses were implemented and the results were visualized with the R packages “clusterProfiler”, “pathview”, “enrichplot”, “ggplot2”, “org.Hs.eg.db”, and “CBNplot”. p < 0.05 indicated a statistically significant difference.

### 2.6 Identification of metabolism-related DEGs

A total of 3937 metabolism-related genes were retrieved from the Human Gene Database GeneCards (https://www.genecards.org/), with the key words “metabolism” and a relevance score > 2.5 as criteria. Similarly, we retrieved 951 metabolism-related genes from the GSEA/MSigDB database (https://www.gsea-msigdb.org/gsea/index.jsp). The results revealed that the two databases shared 780 overlapping genes (**Supplementary Table 2**). By comparing these 780 metabolism-related genes with DEGs and genes in modules related to sPTB (absolute value of correlation coefficient > 0.4 and p < 0.05), we identified 19 candidate hub genes (MRDEGs).

### 2.7 Machine learning to screen hub genes

In this study, we employed three ML algorithms, least absolute shrinkage and selection operator (LASSO), support vector machine recursive feature elimination (SVM-RFE), and random forest (RF) analysis, to further screen hub genes. LASSO logistic regression is a method for building generalized linear models (29). Here, LASSO regression was used to screen variables of MRDEGs with 10-fold cross-validation, and α was set to 1 in these circumstances. SVM-RFE is a backward selection method that starts with all features and then recursively removes the least important features based on model performance. The performance of the model was evaluated using 10-fold cross-validation techniques in this study. The SVM-RFE method provides a feature ranking based on the importance of the features, and the optimal features can be screened to build the final model (30). RF is an ensemble learning method that extends from Bagging. Based on the integration of the decision tree classifier, RF imports random attribute selection in the training of the decision tree, thereby increasing its accuracy and generalizability. We ran the model with 10-fold cross-validation and obtained the genes with the highest accuracy. The LASSO, SVM-RFE, and RF algorithms were implemented through the R packages “glmnet”, “e1071”, and “randomForest”, respectively.

### 2.8 Hub gene validation and nomogram construction

The receiver operating characteristic (ROC) curves and the corresponding area under the curve (AUC) are widely used to assess the classification performance of a model or gene discriminating between samples from a specific patient group and a non-patient group. To verify the performance of the hub genes identified by the three ML algorithms, we generated ROC curves with the R package “pROC” and 8 overlapping hub genes. Moreover, we validated the hub genes in two distinct external datasets (GSE18809 and GSE120480) with ROC curves. Based on the verified hub genes, a nomogram was constructed with the R package “rms”, which can integrate multiple factors as a new classifier to discriminate disease phenotypes. Calibration curves were employed to evaluate the consistency between the observed and predicted values. ROC curves were created to estimate the performance of the nomogram using the internal and external verification datasets.

### 2.9 Building a TF-miRNAs-mRNA regulatory network

NetworkAnalyst (http://www.networkanalyst.ca/faces/home.xhtml) (31) was utilized to predict and establish a TF-miRNA−mRNA network, in which literature curated regulatory interaction information was collected from the TarBase v8.0 and ChEA databases. Subsequently, we analyzed and mapped the results using Cytoscape software to visualize possible upstream and downstream regulatory relationships of genes.

### 2.10 Immune infiltration analysis

Immune infiltration was evaluated through the single-sample gene set enrichment analysis (ssGSEA) algorithm with the R package “GSVA” (32). We compared the distribution of different immune cells between the sPTB and TL groups. Moreover, the correlation between the expression of hub genes and the level of immune cell infiltration was estimated via Spearman correlation analysis.

### 2.11 Immunohistochemistry

All tissues were collected 30 minutes after placental delivery and soaked in 4% paraformaldehyde solution overnight, followed by paraffin embedding. For the sectioning of paraffin-embedded tissue, the tissue was dewaxed, rehydrated and subjected to antigen retrieval before being successively incubated with 3% hydrogen peroxide solution, 3% BSA at room temperature and primary antibodies overnight (**Supplementary Table 5**) at 4°C. The next day, the sections were incubated with secondary antibodies and DAB reagent to stain the tissue.

### 2.12 Statistical analysis

Statistical analysis was performed with RStudio (version 4.1.2) and IBM SPSS Statistics 25 software. The differences in clinical characteristics between sPTB patients and TL patients were analyzed by Student’s t test. P < 0.05 was considered to indicate statistical significance.

## 3 Results

### 3.1 GEO dataset preprocessing and DEGs identification

**Figure 1:**
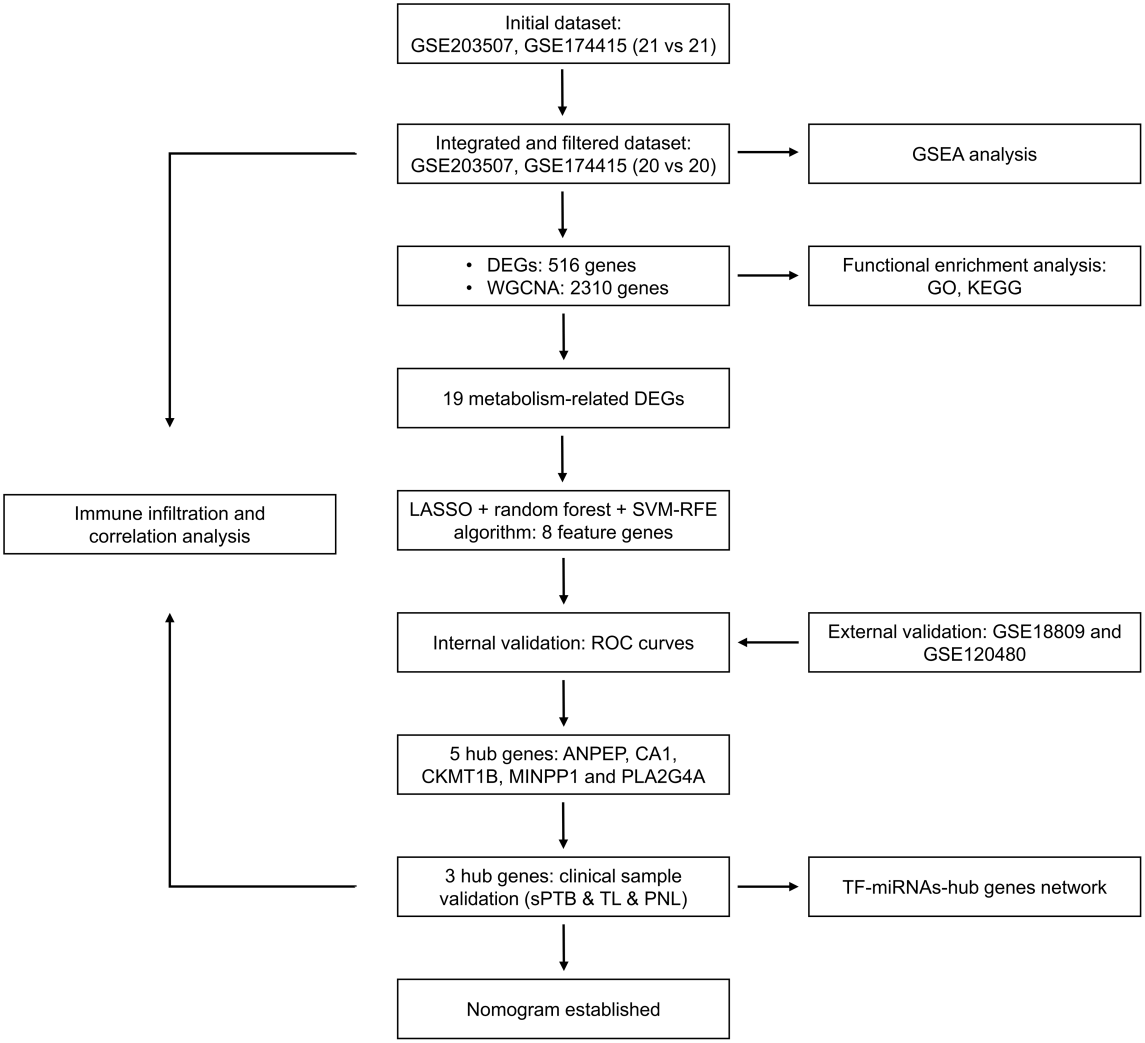
Flow chart of the research design. DEGs, differentially expressed genes; sPTB, spontaneous preterm birth; TL, term labor; PNL, preterm nonlabor.

**Figure 2:**
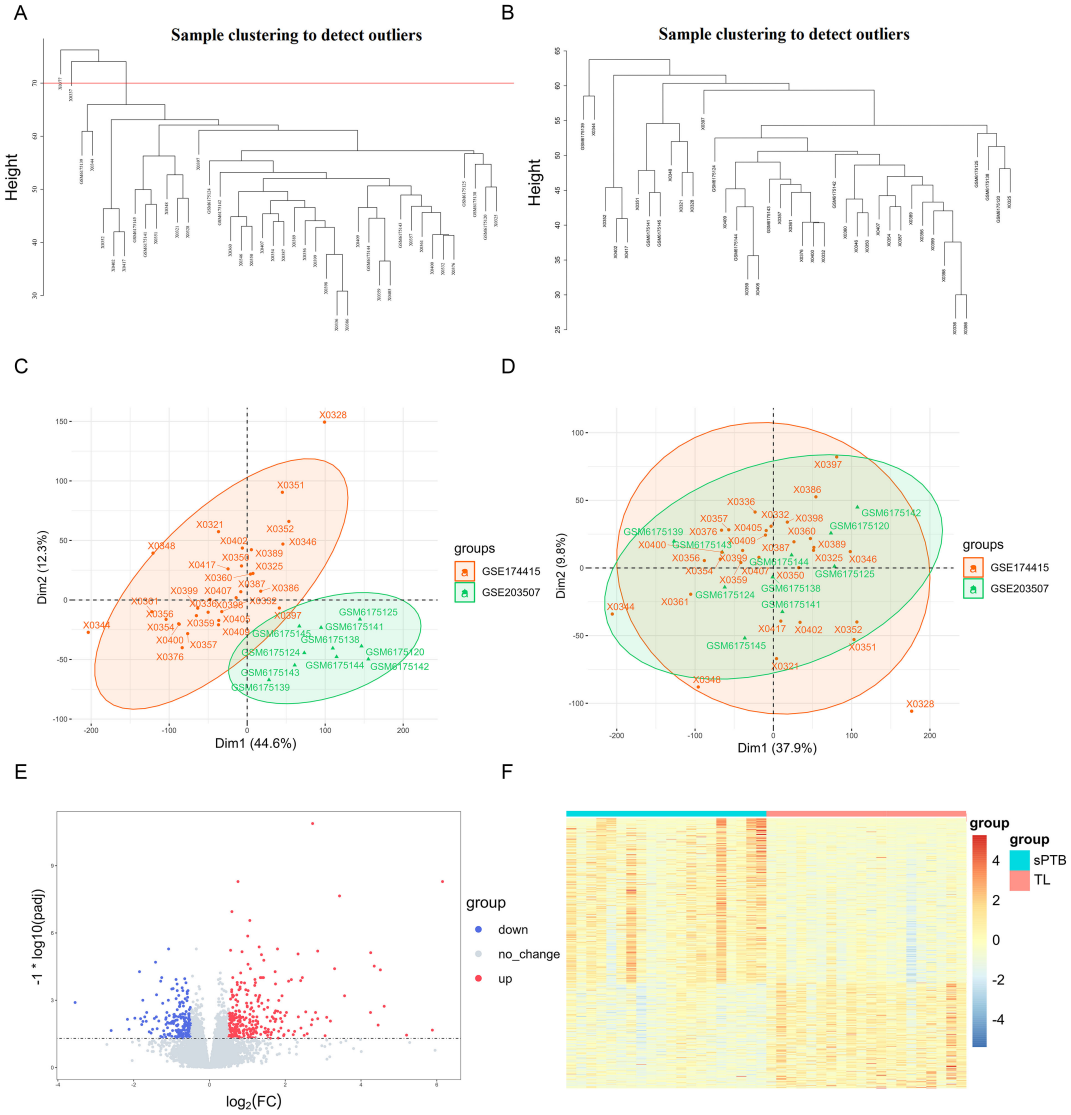
Integration of datasets and DEGs identification **(A)** Cluster dendrogram of the raw data. The region above the red line represents the outlier sample. **(B)** Cluster dendrogram of filtered data. **(C, D)** PCA plots according to merged datasets before and after batch effects were removed. **(E)** Volcano plots of DEGs (p-adjustment < 0.05 as well as log2FC>0.5). **(F)** Heatmap of significant DEGs.

The flow chart for the workflow of this study is shown in **Figure 1**. First, two training sets (GSE203507 and GSE174415) were merged, and batch effects were removed. Then, we identified outlier samples with a cluster dendrogram and eventually removed two samples because their height was greater than 70. **Figures 2A, B** depict the cluster dendrograms of the samples before and after filtering, respectively. We reanalyzed the remaining 40 samples. Before any subsequent analysis, the data were standardized or normalized to eliminate the effect of sequencing depth or differences between samples. **Figures 2C, D** show the principal component analysis (PCA) results of the two datasets before and after the removal of batch effects. The samples from the two datasets were evenly distributed, as shown in **Figure 2D**, indicating that disease differentially expressed gene analysis of the sPTB group and TL group could be conducted. Additionally, box plots and cluster dendrograms of these two datasets before and after de-batching are presented in **Supplementary Figures 1A–D**.

As shown in **Figure 2E**, 307 upregulated genes and 209 downregulated genes were identified as DEGs under the criteria of p-adjusted < 0.05 and log2 | FC | >0.5. The detailed expression of these DEGs is visualized in **Figure 2F**, which shows the differential expression patterns of the sPTB and TL groups intuitively.

### 3.2 WGCNA module identification and correlation analysis

**Figure 3:**
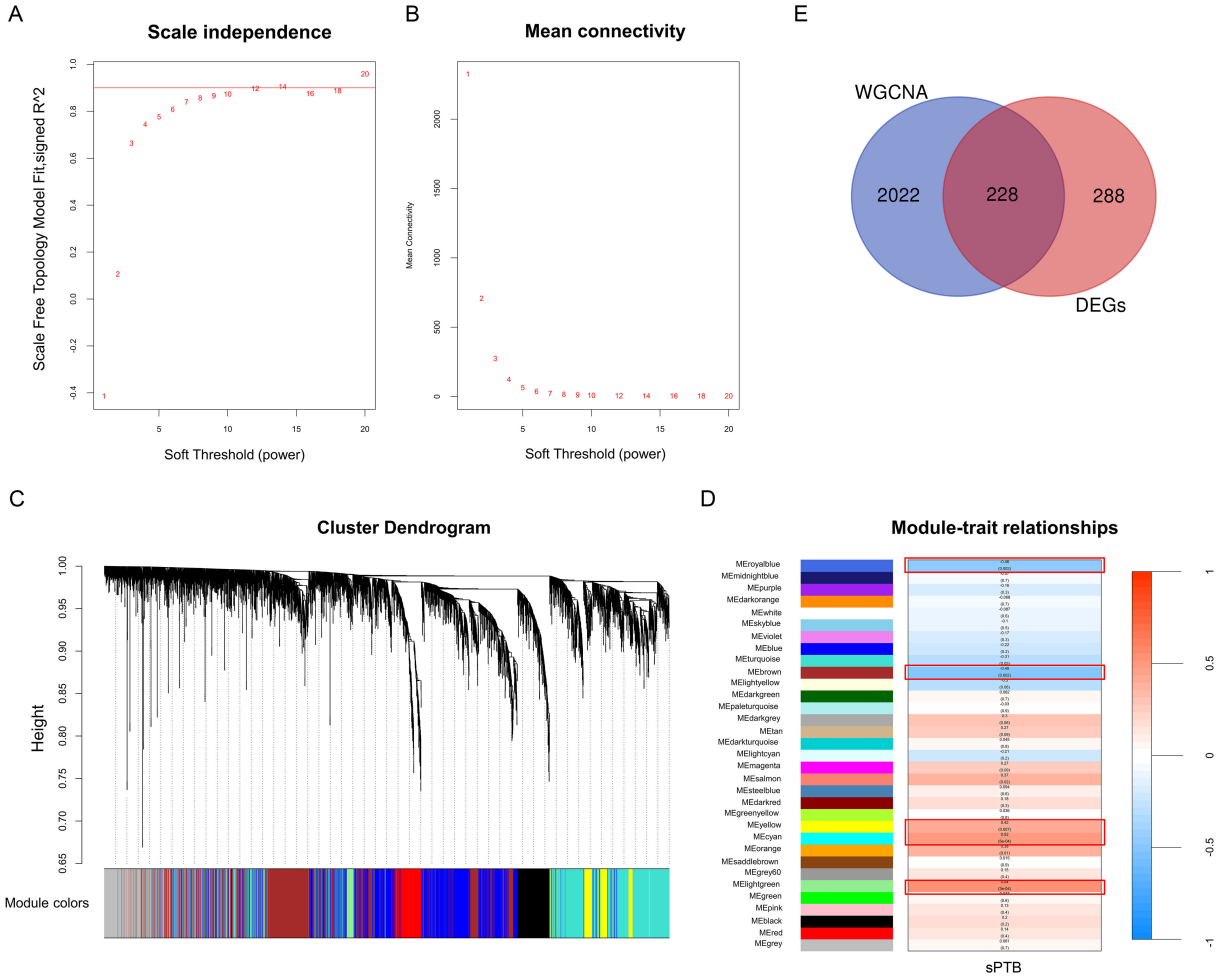
WGCNA **(A, B)** Analysis of network topologies for various soft-threshold powers. **(C)** Clustering dendrogram of coexpressed genes in different modules as indicated by various colors. **(D)** Heatmap of the correlation between module genes and sPTB with the corresponding p values and correlation coefficients. **(E)** Venn diagrams representing the common DEGs and WGCNA modules related to sPTB (p < 0.05 and correlation coefficient > 0.4).

After normalizing the merged datasets (**Supplementary Figure 1E**), we used WGCNA to obtain 32 gene modules whose genes showed similar expression patterns (**Figure 3C**), with the optimal soft threshold value identified as eight to ensure a scale-free distribution of the network (**Figures 3A, B**). Modules with correlation coefficients > 0.4 and p-adjusted < 0.05 were considered significantly correlated with sPTB (**Figure 3D**) and are part of the royal blue, brown-yellow, cyan, and light green modules. Thereafter, these modules were compared with the DEGs to identify 228 sPTB-related DEGs (**Figure 3E**, **Supplementary Table 3**), which were used for subsequent analysis.

**Figure 4:**
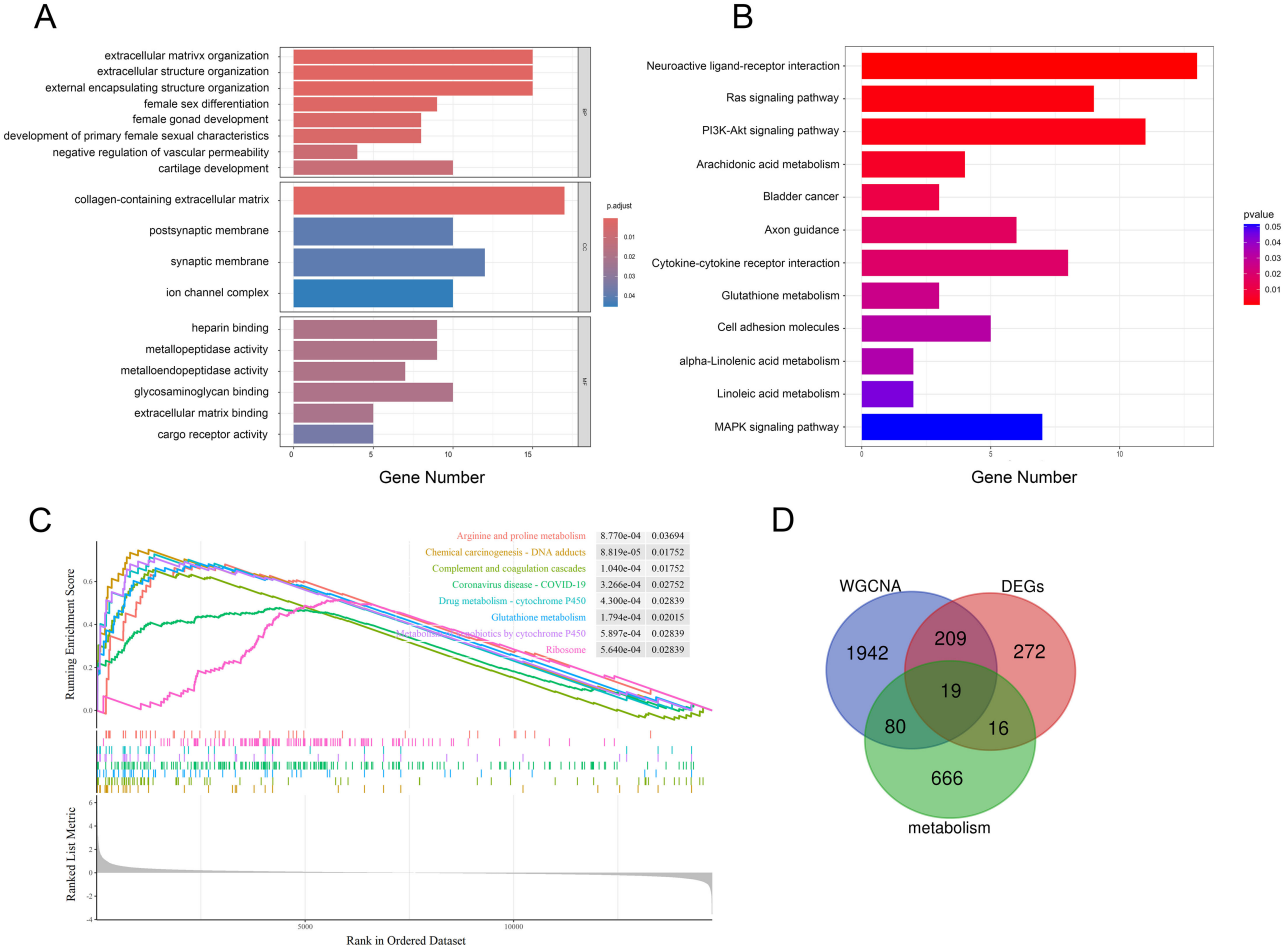
Multiple pathway enrichment analysis. **(A, B)** GO and KEGG enrichment analyses of the shared genes. **(C)** GSEA of sPTB samples. **(D)** Venn diagrams displaying MRDEGs.

We performed functional enrichment of these shared genes via GO and KEGG analyses to further evaluate the potential biological functions of these genes. There categories of GO terms were included in enrichment analysis: biological process (BP), which included extracellular matrix organization and extracellular structure organization; cellular component (CC), which included collagen-containing extracellular matrix; and molecular function (MF), which included metallopeptidase activity and heparin binding, as depicted in **Figure 4A**. KEGG analysis revealed that genes related to neuroactive ligand−receptor interactions, the Ras/PI3K-Akt signaling pathway, cytokine−cytokine receptor interactions and some metabolic pathways, such as arachidonic acid, glutathione, α-linolenic acid and linoleic acid metabolism, were enriched (**Figure 4B**, **Supplementary Figures 2A, B**). GSEA indicated that these two datasets were primarily enriched in glutathione, cytochrome P450 and arginine and proline metabolism (**Figure 4C**). Notably, a number of metabolic pathways enriched in those sPTB samples, which is consistent with what’s been reported before (12, 14). As a medium of communication between mother and fetal, metabolic abnormalities in the placenta may have far-reaching consequences for both sides. Thus, we focused on the metabolic changes in the following analysis and aimed to identify metabolism-related hub genes involved in sPTB. By comparing metabolism-related genes acquired from the open database (**Supplementary Table 2**) and the abovementioned shared genes, we obtained 19 MRDEGs (PAPSS2, CEL, NNMT, SDS, CKMT1B, MINPP1, ASMT, GGT5, LDHD, PLA2G2A, CYP4F3, PLA2G4A, SGPP2, SULT1E1, PDE2A, ANPEP, CA1, GATM, and PGD) to utilize in further analysis (**Figure 4D**). In addition, we speculated that there was a possible relationship between pathways based on GSEA, GO and KEGG with CBNplot analyses (**Supplementary Figure 2**).

### 3.3 Identification and validation of hub MRDEGs with three machine learning algorithms and IHC

**Figure 5:**
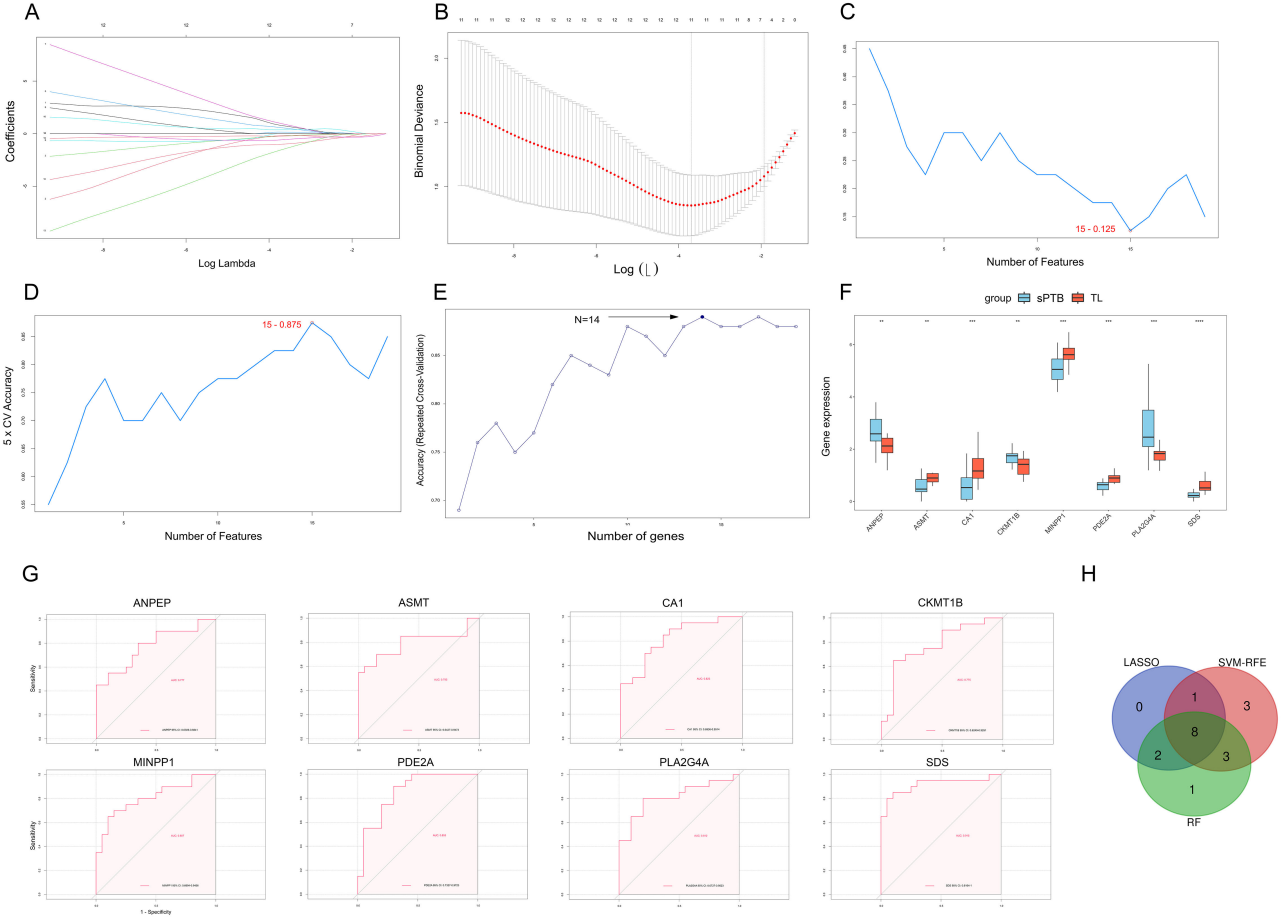
Three machine learning methods were utilized to identify hub genes. **(A)** LASSO coefficient diagram. Each curve represents one specific gene. **(B)** The cross-validation curve shows the best λ value, and the X-axis above corresponds to the number of variables. **(C, D)** Visualization of the SVM-RFE results demonstrates that the accuracy and error of the model vary with the number of features. The features with the highest accuracy and lowest error were used for subsequent studies. **(E)** RF analysis was used to identify the genes with the highest accuracy. **(F)** Boxplot showing the differential expression of 8 genes. (**p < 0.01; ***p < 0.001; ****p < 0.0001, Student’s t test). **(G)** The ROC curves of ANPEP, ASMT, CA1, CKMT1B, MINPP1, PDE2A, PLA2G4A, and SDS. **(H)** Venn diagram depicting the candidate hub genes obtained by comparing the results of three machine learning algorithms.

To further identify feature genes that could play a role in sPTB, we used the LASSO, RF, and SVM-RFE algorithms to identify hub genes based on the MRDEGs identified above. The LASSO algorithm extracted 11 genes from the MRDEGs (**Figures 5A, B**). Based on SVM-RFE, we identified 14 genes by tenfold cross-validation (**Figures 5C, D**). In RF, the optimal number of trees was defined as 14 when the error rate was minimized (**Figure 5E**). The results of the three ML algorithms shared 8 feature genes, namely, ANPEP, ASMT, CA1, CKMT1B, MINPP1, PDE2A, PLA2G4A and SDS (**Figure 5H**), which were regarded as candidate hub genes in sPTB. The different expression patterns of the 8 significant MRDEGs in the sPTB and TL groups are depicted in **Figure 5F**.

To validate the contribution of these 8 MRDEGs to differentiation of the sPTB and TL groups, ROC analysis was utilized for the training sets and validation sets. In the training sets, which included the combined GSE203507 and GSE174415 datasets, all 8 MRDEGs had area under the curve (AUC) values exceeding 0.75, as depicted in **Figure 5G**. Then, we performed ROC analysis on two external validation datasets (GSE18809 and GSE120480) separately. Three genes (ASMT, PDE2A and SDS) were excluded because their AUC values were less than 0.75 (**Supplementary Figures 3A**, **4A**). Finally, we selected 5 MRDEGs (ANPEP, CA1, CKMT1B, MINPP1 and PLA2G4A) as feature hub genes in sPTB.

**Figure 6:**
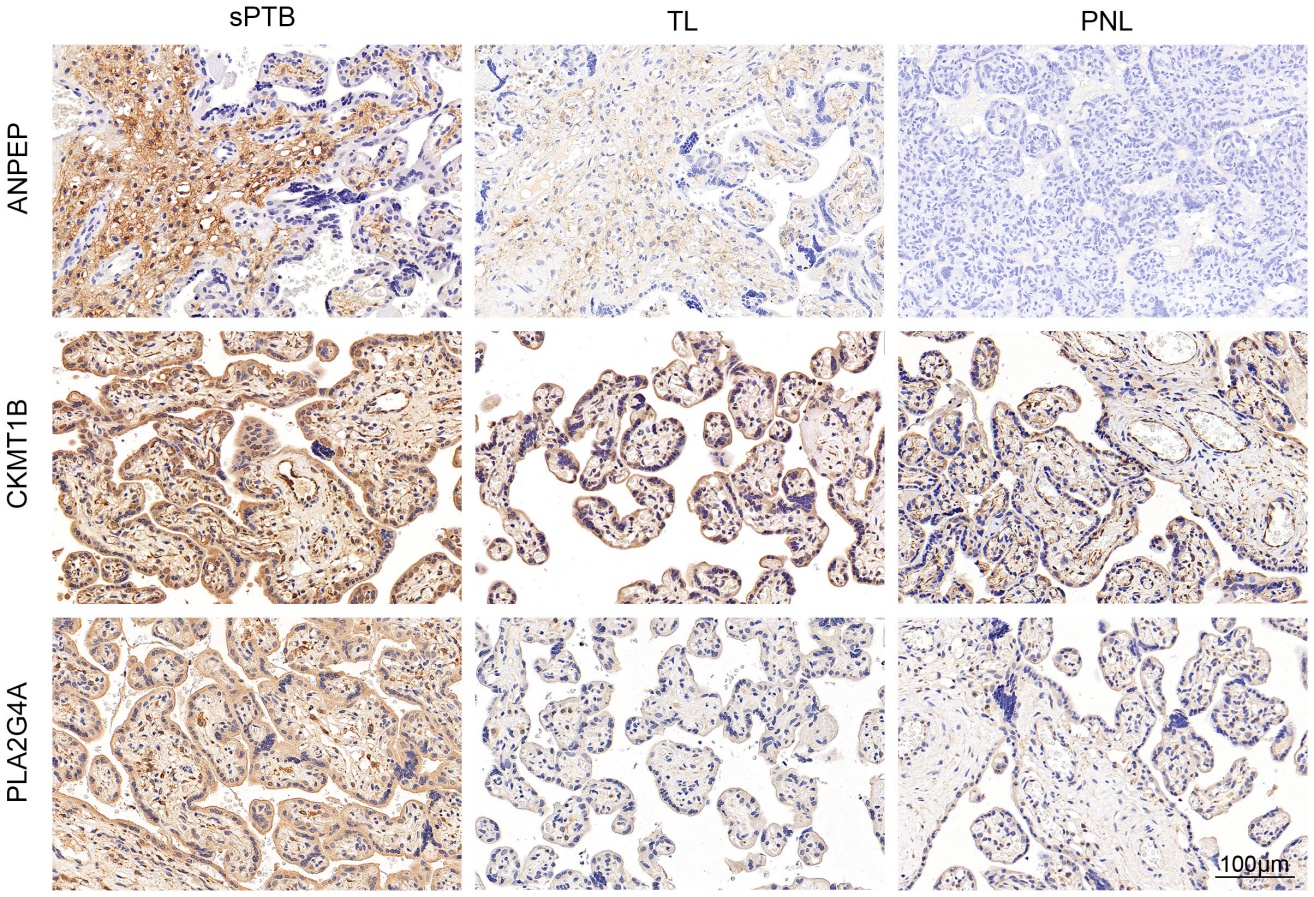
Validation of metabolism-related hub genes by IHC. IHC staining verified the protein expression of the hub genes.

To verify the pivotal roles of the hub genes (ANPEP, CA1, CKMT1B, MINPP1 and PLA2G4A), we performed immunohistochemical staining of placentas from the sPTB and TL groups (n=5). As shown in **Figure 6** and **Supplementary Figures 5**, **6**, the levels of ANPEP, CKMT1B and PLA2G4A were significantly greater in the sPTB group than in the TL group, which was consistent with the results of the bioinformatics analysis. Notably, unlike the other four genes located in trophoblast cells, ANPEP was mainly expressed in intravillous stromal cells. MINPP1 was barely expressed in the placenta; therefore, we excluded it from the list of hub genes.

Additionally, to confirm that the differences in the expression of these hub genes were caused by pathological changes rather than variations between term and preterm births due to gestational age (GA), we compared the expression levels with 37 previously identified GA-specific candidate genes (33) and evaluated the differences in expression between the placentas of sPTB patients and preterm non-labor (PNL) patients via immunohistochemistry (**Figure 6**; **Supplementary Figures 5**, **6**, n=3). Ultimately, we regarded CA1 as a GA-related gene because of its low and undifferentiated expression in both the PNL and sPTB groups and its high expression in the TL group. Hence, we eliminated CA1 from the list of hub genes. Ultimately, we kept ANPEP, CKMT1B, and PLA2G4A as the hub genes, which were enriched in glutathione (GSH) and arachidonic acid metabolism. Both of them are reported to be involved in the development of sPTB (12, 34).

### 3.4 The establishment of nomograms and the TF-miRNA-hub gene network

**Figure 7:**
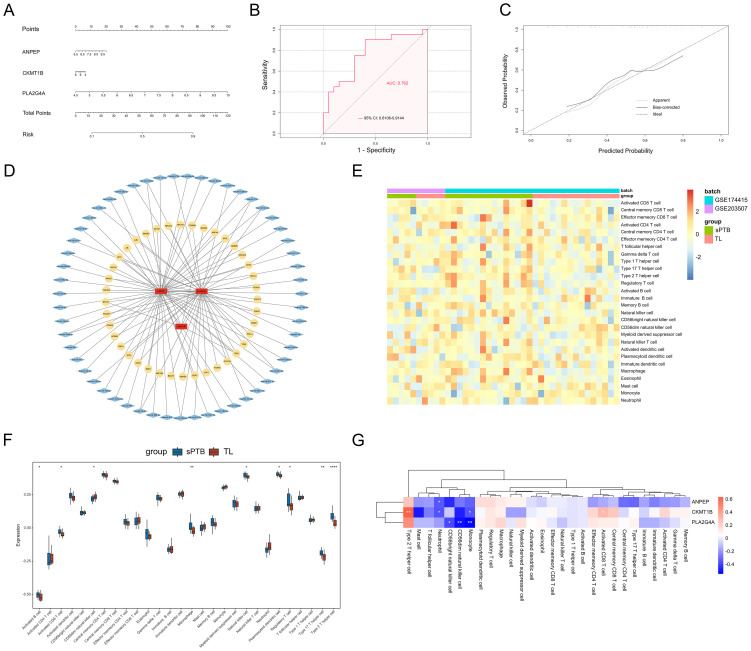
Predicted functional network and immune infiltration analysis. **(A)** Nomogram of 3 hub genes. **(B)** The ROC curves of the above nomograms. **(C)** Calibration curve. **(D)** The predicted TF-miRNA-hub gene network. **(E)** Heatmap of immune infiltration profiles. **(F)** Differential infiltration of immune cells. (*p < 0.05; **p < 0.01; ****p < 0.0001, Student’s t test). **(G)** Correlations between the 3 hub genes and immune cells. (*p < 0.05; **p < 0.01; ****p < 0.0001, Spearman correlation analysis).

To determine whether these hub genes have a comprehensive effect on sPTB, we established a nomogram based on the expression of the remaining 3 hub MRDEGs in the training set (**Figure 7A**) and validated the results using the GSE18809 and GSE120480 datasets. ROC curves and calibration curves were generated, which indicated that the nomogram could accurately evaluate the risk of sPTB (**Figures 7B, C**). In addition, the nomogram also fit the GSE18809 and GSE120480 datasets, which confirmed the critical role of the 3 hub genes (**Supplementary Figures 3B, C**, **4B, C**).

To investigate the upstream regulatory factors of the 3 hub genes, we established a TF-miRNA-hub gene network based on NetworkAnalyst. The network included 40 TFs and 55 miRNAs. In addition, PLA2G4A and ANPEP can be regulated by some mutual miRNAs, and they both shared one upstream TF, TP63. **Figure 7D** shows the relationships between the 3 hub genes and their potential TFs and miRNAs in detail.

### 3.5 Immune cell infiltration analysis

The immune cells that exist in the basal plate of the placenta (decidua) have been extensively studied during placental implantation and development, as well as parturition (35). However, the quantity and function of immune cells in the interstitial space within the placental villi are poorly understood. We used ssGSEA to determine the differences in the infiltration of 22 immune cell types between the sPTB and TL groups. **Figure 7E** illustrates the diverse distribution of immune cells in each sample. Activated B cells, activated CD8 T cells, natural killer cells, macrophages, Th2 cells, Tregs and Th17 cells were significantly increased in sPTB patients (**Figure 7F**), indicating that the modifications in the immune microenvironment may contribute to the development of sPTB. Then, we calculated the correlations between the hub genes and immune cell infiltration in the placenta and found that the hub genes were related to NK cells, Th2 cells, activated CD8^+^ T cells, monocytes and neutrophils (**Figure 7G**). Among the immune cells, PLA2G4A was most strongly related to NK cells and monocytes, while ANPEP and CKMT1B were correlated with neutrophils, indicating that metabolism may influence immune cell infiltration to some extent. This finding suggested a potential mechanism of sPTB and future directions of treatment.

## 4 Discussion

Preterm birth, as a syndrome with profound impact, places a great burden on the health of premature infants and the financial conditions of their families. By understanding and exploring its pathogenesis, we can prevent the occurrence of preterm birth and intervene early. Accumulating evidence suggests that metabolism has an impact on the occurrence of sPTB. Several studies suggested that maternal metabolic disorders such as bile acid (BA) (36), folate (37) and glucose (38) metabolism disorders and dyslipidemia (39–41) were associated with increased risks of preterm birth. Besides, many metabolic disorders were observed in placenta of sPTB as mentioned before. Placenta is an important organ for communication between mother and fetus. Normal placental development and stable placental function are essential for a successful pregnancy. While metabolic disorders may disrupt homeostasis, leading to early senescence, inflammation, and oxidative stress in the placental (19, 42–46). Therefore, identifying the metabolic characteristics of placenta in sPTB patients may provide a new perspective on treatment and improve patient prognosis. To more precisely determine the role of placental metabolic abnormalities in sPTB, we excluded patients with known maternal metabolic disorders.

In this study, we integrated two GEO datasets, GSE203507 and GSE174415, which included 20 sPTB samples and 20 TL samples collected from human placentas as the training set. After identifying sPTB-related genes by comparing WGCNA and DEGs, we carried out pathway enrichment analysis; the results revealed that several metabolism-related pathways, such as arachidonic acid metabolism, glutathione metabolism, and α-/linolenic acid metabolism, were involved. Previous studies of placental transcriptomic and metabolome signatures revealed that impaired placental bioenergetic and metabolic stability may be involved in sPTB (12, 47, 48). However, the molecular mechanisms that cause metabolic changes are still unknown. In this study, we used multiple machine learning methods to identify metabolism-related hub genes in sPTB and focused on the pathways associated with those genes.

We noticed that the hub genes we identified were mostly enriched in glutathione (GSH) and arachidonic acid metabolism. GSH metabolism was enriched in both the GSEA and KEGG analyses and was associated with the clearance of reactive oxygen species (ROS). In a reaction catalyzed by glutathione peroxidase (GSH-PX), GSH reduces intracellularly produced H_2_O_2_ to O_2_ and H_2_O, while at the same time, GSH is oxidized to glutathione (GSSG), which is catalyzed by glutathione reductase (GSR) to regenerate GSH (49, 50). Through this process, GSH can remove oxygen ions and other free radicals in the body and inhibit lipid peroxidation. Some studies have suggested that oxidative stress generation and GSH are involved in sPTB (12, 51, 52), and that glutathione-mediated detoxification is disrupted in sPTB placentas. KEGG analysis revealed that the metabolism of arachidonic acid (AA) was also altered in sPTB and TL patients; this result was also reported in another transcriptomic analysis (14). In general, AA derivatives are referred to as eicosanoids that are generated through three different metabolic pathways and are widely involved in multiple physiological and pathological processes (53, 54). Through the cyclooxygenase (COX), lipoxygenase (LO), and cytochrome P450 (CYP450) pathways, AA can be converted into prostaglandins (PGs), leukotrienes (LTs), epoxyeicosatrienoic acids (EETs) or hydroxyeicosatetraenoic acids (HETEs) (55, 56). PGE2 and HETEs synthesized from arachidonic acid can facilitate the ripening of the cervix, myometrial contraction and membrane activation during parturition and preterm labor (34, 57, 58); the levels of PGE2 and HETEs were significantly increased in sPTB placentas (12). Regarding infection-related preterm birth, the level of arachidonate lipoxygenase metabolite leukotriene B4 (LTB4) was markedly increased in the placenta and amniotic fluid (59, 60). Moreover, higher concentrations of 5-HETE or DHA signal a greater risk of spontaneous preterm birth (61), thus confirming the role of AA metabolism in sPTB.

Based on the enriched metabolism-related pathways, we wondered whether hub genes in sPTB placentas are involved in those pathways. Data from the Human Gene Database and GeneCards were combined with that from the GSEA/MSigDB database to identify metabolism-related genes. Then, we employed three ML algorithms to acquire 8 candidate hub genes. ML is a subset of artificial intelligence with the goal of enabling machines to perform tasks that require human intelligence, such as diagnosis, planning, and prediction. Based on existing data, ML can reveal potential models of gene expression in different groups and then make predictions to identify critical genes that are effective targets for disease treatment. In our study, 8 genes (ANPEP, ASMT, CA1, CKMT1B, MINPP1, PDE2A, PLA2G4A and SDS) were identified by the three ML methods (LASSO, RF, and SVM-RFE); then, we utilized ROC curves to verify their ability to distinguish the sPTB and TL groups in the training and validation sets. In this way, 5 hub genes (ANPEP, CA1, CKMT1B, MINPP1 and PLA2G4A) that may play principal roles in sPTB through metabolic processes were identified.

Given the difficulty of obtaining placental samples from the same individual at different times of gestation, exploring longitudinal changes in placental metabolism with gestational weeks in humans is hard to achieve. However, several studies of the blood composition have shown possible differences in maternal metabolism across gestational weeks, including lipid metabolism (62), carbon metabolism (63), glucose metabolism (64) and so on. Also, a study elucidated the characterization of normal longitudinal changes in placental metabolism throughout pregnancy in mice (65). Therefore, considering the possible effect of different gestational ages on transcription levels in the TL and sPTB groups, we included another group, the PNL group, in the validation process. Ultimately, we identified 3 metabolism-related genes (ANPEP, CKMT1B and PLA2G4A) as hub genes.

ANPEP (APN, CD13) is an enzyme that is capable of releasing an N-terminal amino acid from a peptide, amide or arylamide and is enriched in the GSH metabolism pathway. ANPEP is involved in the processing of various peptides, including peptide hormones, such as angiotensin III and IV, and may play a role in angiogenesis (66), decidualization, and placental implantation (67, 68). In late pregnancy, the expression of ANPEP is significantly lower at E20d in mouse placenta (69), which is different from the high expression in sPTB in our study. CKMT1B is related to creatine and Inositol phosphate metabolism (70, 71), and CKMT1B deficiency may be related to preterm birth and neurodevelopmental defects in newborns (72). However, the expression and distribution of ANPEP and CKMT1B in the placenta have not yet been characterized by other study. PLA2G4A (cPLA2-Alpha) is a member of the cytosolic phospholipase A2 group IV family that catalyzes the hydrolysis of membrane phospholipids to release arachidonic acid, which is subsequently metabolized into eicosanoids, mainly prostaglandins (73). Previous studies have shown that a lack of PLA2G4A leads to deferred implantation; this effect can be reversed by exogenous PG treatment (74). However, the effect of increased PLA2G4A expression on pregnancy and labor has not yet been studied. As pregnancy progresses, the expression of PLA2G4A increases in the mouse placenta (75). In other mammals, the same phenomenon was observed in multiple gestational tissues, such as the fetal membrane (76), decidua and myometrium (77), which facilitated the initiation of labor. Additionally, the accumulation of the PLA2G4A protein was confirmed in the human fetal membrane and decidua (78), and the presence of PLA2G4A in the human placenta has been confirmed (79). Considering the increased expression of PLA2G4A and arachidonic acid metabolism in sPTB patients in our study, we assumed that PLA2G4A may contribute to sPTB through AA metabolism and the generation of PGs.

The immune environment at the maternal-fetal interface has received much attention in recent years. We investigated immune cells that may promote sPTB in the placenta and found that macrophages, CD8^+^ T cells, Th2 cells, and Th17 cells exhibited strong heterogeneity between the sPTB and TL groups. Macrophages are considered indispensable for placenta formation and the maintenance of maternal-fetal tolerance in early pregnancy (80). Macrophage infiltration and M1-like macrophage polarization are observed in sPTB patients (81), and these cells release the inflammatory cytokines NF-κB, TNF, and IL-10 to initiatedelivery (82). Evidence suggests that enriched activated T cells cause pathological inflammation at the maternal-fetal interface, leading to preterm labor (83). At present, there is no precise evidence showing that Th17 and Th2 cells are involved in the occurrence of sPTB. However, Th17 cell-mediated local inflammation can directly induce fetal loss *in vivo (* 84).

There are also several limitations to our study. First, our analysis included only 40 samples, which may increase the variation in the estimated correlation coefficient and reduce the repeatability of the correlation coefficient. Additionally, a small sample size could result in overfitting or underfitting the target tasks in machine learning with limited samples. To minimize the impact of this problem, we combined the results of three ML models (LASSO, SVM-RFE, and RF) and conducted internal and external validation to increase the reliability of our results. Second, the differential expression of genes was only verified in clinical samples at the protein level with IHC. More relevant experiments should be carried out in the future with the isolated specific cells in clinical samples. Moreover, the specific roles of these genes in the occurrence and development of sPTB should be explored, as well as their upstream molecular regulatory mechanism, which we will investigate in future studies. Third, the data we analyzed were derived from bulk RNA-seq, in which the expression of genes was affected by different types of cells contained in the sampled tissue. To address the whole-field challenge, single-cell RNA sequencing and single-cell metabolomics are recommended, given their ability to provide single-cell resolution and to detect intercellular communication. Fourth, patients with inherited metabolic disorders and any clinical diagnosis of metabolic disorder were excluded in this study. This may limit the comprehensiveness of the study. Although we’d rather focused on the placental metabolic disorder appeared in sPTB in this study, preterm birth associated with maternal and genetic metabolic abnormalities still deserved attention and research. In addition, another focus of future research should be sequencing based on the subtype of preterm birth. Different subtypes of sPTB could have disparate origins, and some research has initially confirmed this hypothesis (14).

Overall, through intensive and comprehensive bioinformatics analysis, we identified the potential roles of metabolism in sPTB. Additionally, we identified 3 metabolism-related hub genes that are involved in the pathological process of sPTB, namely, ANPEP, CKMT1B and PLA2G4A. Using these hub genes, we constructed a nomogram and performed ROC curve analysis to confirm the influence of the hub genes on the occurrence of spontaneous preterm birth. These findings may offer practical molecular targets for further study, as well as the development of potential therapeutic methods for prevention and intervention.

## Data Availability

The original contributions presented in the study are included in the article/**Supplementary Material**. Further inquiries can be directed to the corresponding author.
