# Supplementary material for: Identification of key metabolism-related genes and pathways in spontaneous preterm birth: combining bioinformatic analysis and machine learning
Source: Front Endocrinol (Lausanne). 2024 Aug 20;15:1440436. doi: 10.3389/fendo.2024.1440436 (PMC11368757; doi:10.3389/fendo.2024.1440436)
Supplement: Supplementary file 1 [file DataSheet1.pdf]

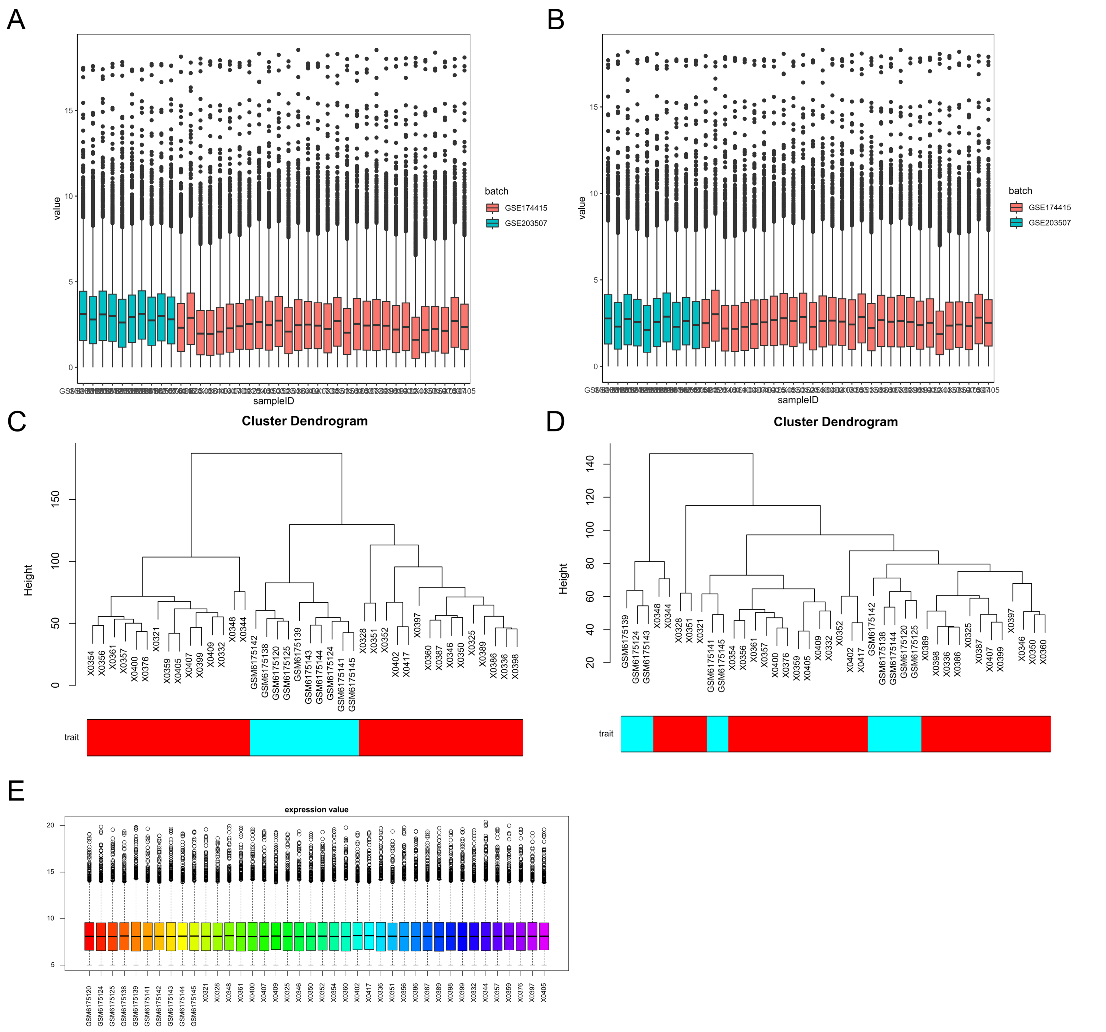

Supplementary Figure 1 The combination of two training datasets.

(A, B) Cluster dendrograms of the merged dataset before (A) and after (B) batch effects were removed. (C, D) Box plots of the merged dataset before (C) and after (D) batch effects were removed. (E) Box plots of the merged dataset after normalization.

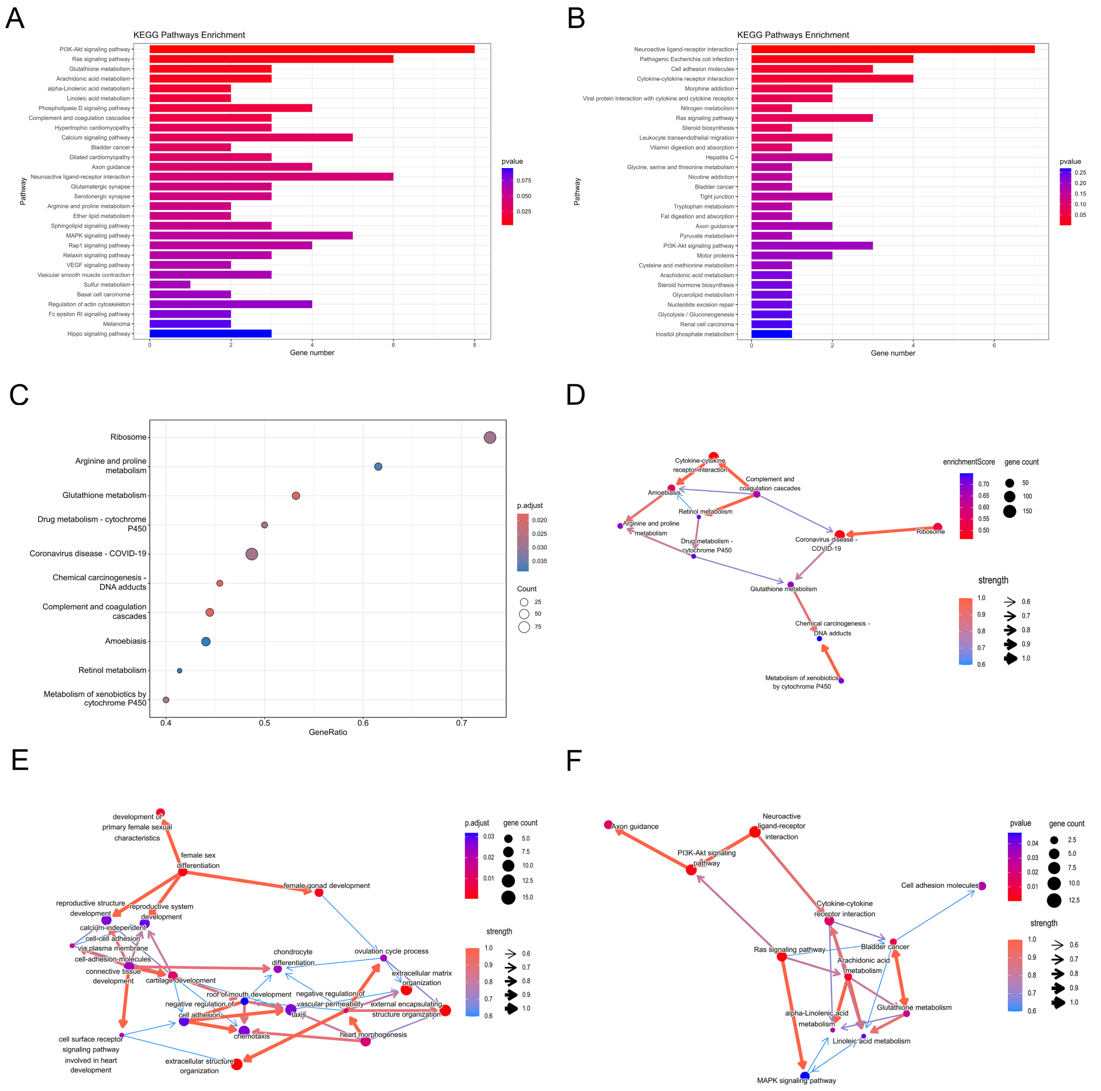

Supplementary Figure 2 Functional enrichment analysis.

(A, B) KEGG analysis of upregulated (A) and downregulated (B) genes. (C) GSEA of sPTB patients. (D-F) CNB plot analysis based on GSEA, GO and KEGG.

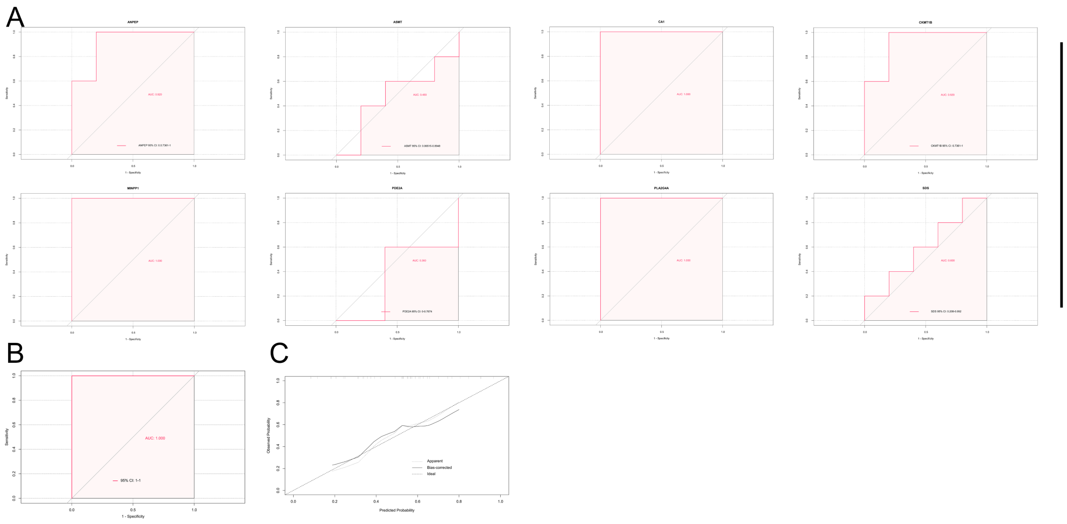

Supplementary Figure 3. Verification via the validation set GSE18809.

(A) The ROC curves of ANPEP, ASMT, CA1, CKMT1B, MINPP1, PDE2A, PLA2G4A, and SDS. (B) The ROC curves of the above nomogram. (C) Calibration curve.

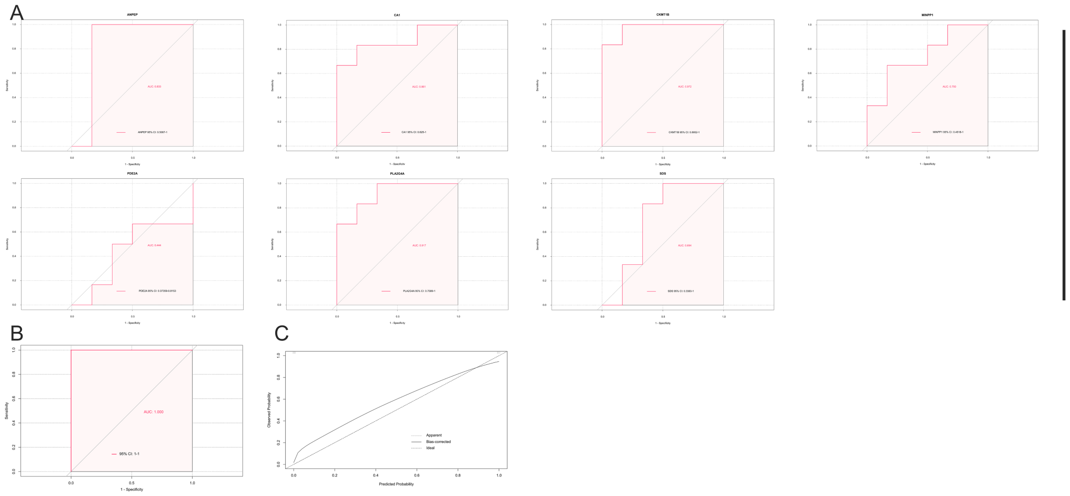

Supplementary Figure 4 The results were verified in the validation set GSE120480.

(A) The ROC curves of ANPEP, CA1, CKMT1B, MINPP1, PDE2A, PLA2G4A, and SDS. (B) The ROC curves of the above nomogram. (C) Calibration curve.

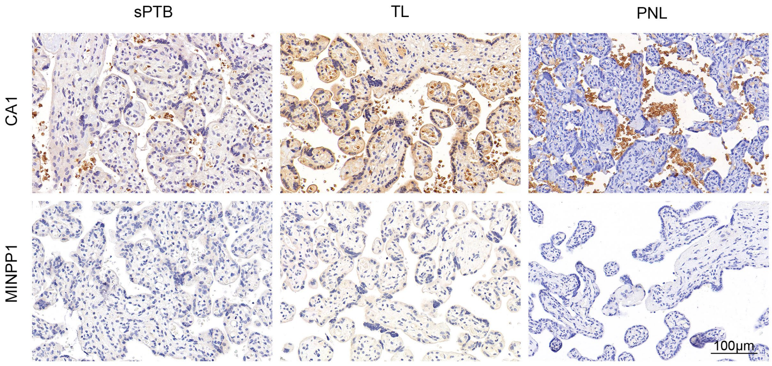

Supplementary Figure 5 Validation of CA1 and MINPP1 expression by IHC.

IHC staining verified the protein expression of CA1 and MINPP1 (sPTB&TL group, n=5; PNL group, n=3).

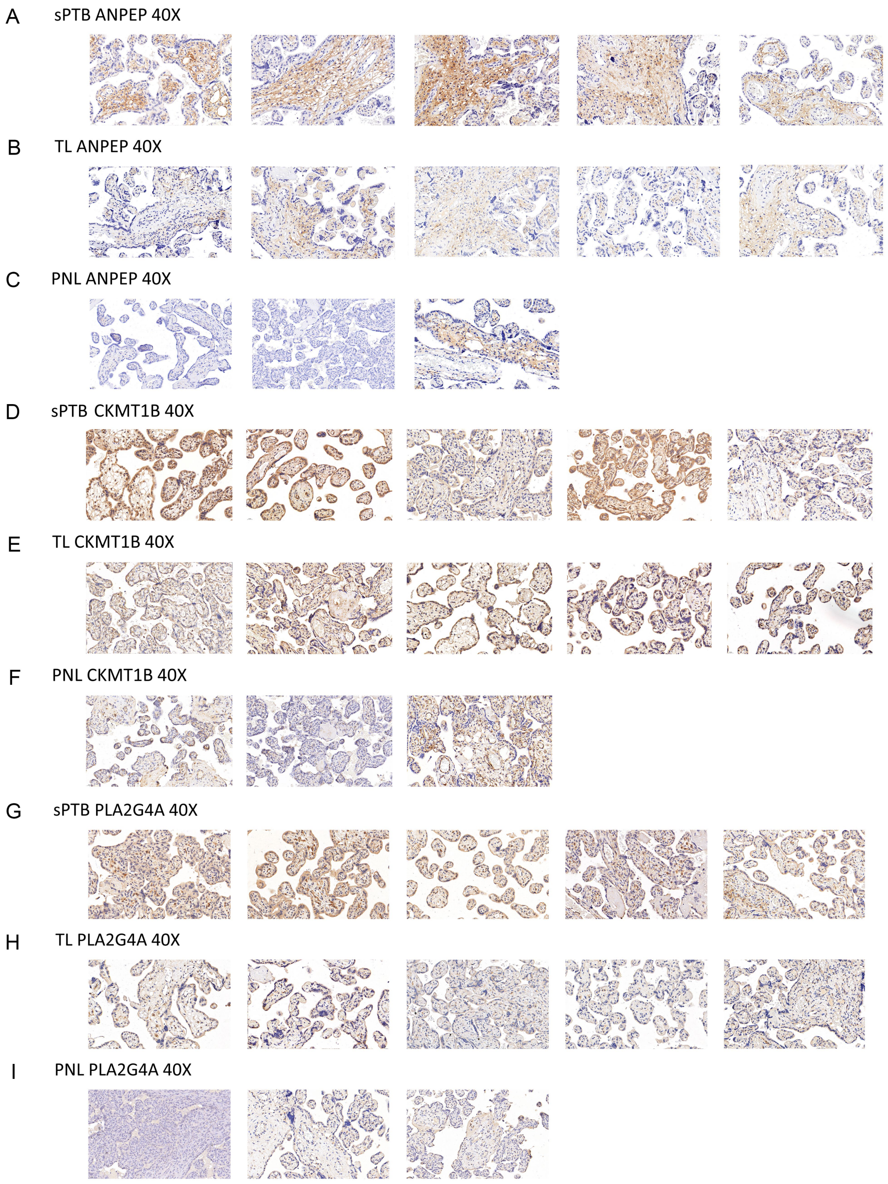

Supplementary Figure 6 Validation of ANPEP, CKMT1B, and PLA2G4A expression by IHC.

IHC staining verified the protein expression of ANPEP, CKMT1B, and PLA2G4A (sPTB&TL group, n=5; PNL group, n=3).
